# Mechanisms of Mixed Th1/Th2 Responses in Mice Induced by *Albizia julibrissin* Saponin Active Fraction by *in Silico* Analysis

**DOI:** 10.3390/vaccines8010048

**Published:** 2020-01-27

**Authors:** Jing Du, Junjie Jin, Juanjuan Wang, Hongxiang Sun

**Affiliations:** 1 Key Laboratory of Animal Virology of Ministry of Agriculture, College of Animal Sciences, Zhejiang University, Hangzhou, Zhejiang 310058, China; 11317036@zju.edu.cn (J.D.); 2013038@wzvcst.edu.cn (J.J.); jjwang@asieris.cn (J.W.); 2 College of Animal Sciences, Wenzhou Vocational College of Science and Technology, Wenzhou 325006, China

**Keywords:** *Albizia julibrissin* saponin, adjuvant, Newcastle disease virus-based recombinant influenza vaccine, adaptive immunity, transcriptome and proteome, bioinformatics

## Abstract

The purified active fraction of *Albizia julibrissin* saponin (AJSAF) is an ideal adjuvant candidate that improves antigen-specific both cellular and humoral immune responses and elicits mixed Th1/Th2 responses, but its mechanisms remain unclear. The key features of action of AJSAF were investigated in mice immunized with Newcastle disease virus-based recombinant influenza vaccine (rL-H5) and AJSAF at the same leg (AJSAF+rL-H5) or different legs (AJSAF/rL-H5). The adjuvant activity of AJSAF on rL-H5 is strictly dependent on their spatial colocalization. Serum H5 antigen (H5Ag)-specific IgG, IgG1, IgG2a, and IgG2b antibody titers in AJSAF+rL-H5 group were significantly higher than those in AJSAF/rL-H5 group. The mechanisms of selectivity of Th1 or Th2 in mice induced by AJSAF was explored by the transcriptomic and proteomic profiles of H5Ag-stimulated splenocytes from the immunized mice using gene microarray and two-dimensional difference gel electrophoresis coupled with matrix-assisted laser desorption/ionization time-of-flight mass spectrometry. Compared to rL-H5 alone, AJSAF/rL-H5 induced more differentially expressed genes (DEGs) than AJSAF+rL-H5, whereas AJSAF+rL-H5 upregulated higher mRNA expression of Th1 (T-bet, IFN-γ, TNF-α, IL-12β, and IL-12Rβ1) and Th2 (IL-10 and AICDA) immune response genes. The neutrophil response and its derived S100A8 and S100A9 might be involved in the AJSAF-mediated Th1 response. Meanwhile, AJSAF might induce the adaptive immune responses by improving a local innate immune microenvironment. These findings expanded the current knowledge on the mechanisms of action of saponin-based adjuvants, and provided new insights into how adjuvants shape adaptive immune responses.

## 1. Introduction

Adjuvants are essential components of new generation vaccines. Adjuvants not only augment the adaptive immune response to vaccines, but also induce the most effective immune response types for specific pathogens. Th1 or Th2 responses generated upon antigenic stimulation can be modulated in vivo depending on the adjuvant used for immunization [1]. The Th1 immunity, correlated with the cellular immune response, is required for therapeutic cancer vaccines, as well as vaccines directed against intracellular pathogens such as viruses, certain bacteria, and parasite [2]. The Th2 immunity, which controls the humoral immune response, is effective for protection against extracellular pathogens including most bacteria and certain viruses [3]. The Th1/Th2 paradigm provides a useful model for understanding the mechanisms of adjuvant and the basis for the rational design of new adjuvants.

How the nature of adjuvants determines T-cell response type is an area of great interest, and the mechanisms responsible for this regulation are only presently being unraveled. The adjuvants are usually classified into pattern recognition receptor (PRR)-dependent and -independent types. An increasing number of studies have focused on pathogen-associated molecular patterns (PAMPs) as candidate Th1 adjuvants, which were recognized by PRRs especially toll-like receptors (TLRs) to activate dendritic cells (DCs) resulting in the generation of IL-12p70 or interferons (IFNs) critical for the Th1 polarization [4]. 3-*O*-desacyl-4’-monophosphoryl lipid A (MPL), a TLR4 ligand, efficiently induced DC maturation and further enhanced Th1 responses through IL-12p70 [5]. PRR-independent adjuvants such as Alum and MF59 were empirically used and have been proved to be effective adjuvants, whereas incomplete understanding of their mechanisms has seriously hampered further development. The most widely used adjuvant Alum was reported to exert adjuvant activity through inducing NLRP3 inflammasome [6,7,8] and damage-associated molecular patterns (DAMPs) such as uric acid (UA) [9] and host DNA [10,11]. However, some studies showed their limited roles in Alum adjuvant activity or even remained contradicted with other reports [12,13]. Therefore, although there have been some reports on the mechanism of Th1 or Th2 selectivity, the details concerning integrated mechanisms of action of adjuvants remain unclear.

The purified active fraction of saponin from the stem bark of *Albizia julibrissin* Durazz. (AJSAF) would be a promising adjuvant candidate for vaccines. It has been proved to improve antigen-specific cellular and humoral immune responses, and simultaneously elicit mixed Th1/Th2 responses in mice to the H5 avian influenza vaccine [14] and porcine reproductive and respiratory syndrome virus vaccine [15]. In our previous studies, it was found that the colocalization of AJSAF with antigen or not significantly affected its adjuvant activity in mice. In fact, the adjuvant activities of other adjuvants such as AS03, chitosan, and phytol derivatives were also reported to depend on their spatial and temporal colocalization with the antigen [16].

In this study, the effects of the colocalization of AJSAF with antigen or not on its adjuvant activity were investigated in mice using the Newcastle disease virus-based recombinant influenza vaccine (rL-H5). Further, the mechanisms resulting in the differences of antigen-specific immune responses between two injection regimens were explored using gene microarray and two-dimensional difference gel electrophoresis coupled with matrix-assisted laser desorption/ionization time-of-flight mass spectrometry (2D DIGE–MALDI-TOF-MS).

## 2. Materials and Methods

### 2.1. Materials

Newcastle disease virus (NDV)-based recombinant influenza vaccine (rL-H5) and H5 subtype AIV hemagglutination inhibition detecting antigen (H5Ag) were purchased from the Harbin Weike Biotechnology Development Co., Heilongjiang, China. RPMI medium was from Hyclone/GE Healthcare, Logan, UT, USA; fetal bovine serum (FBS) was from Gibco, Grand Island, NY, USA. Rabbit anti-mouse IgG peroxidase conjugate were purchased from Sigma Chemical Co., St. Louis, MO, USA; goat anti-mouse IgG1 and IgG2b peroxidase conjugates were from Southern Biotech. Assoc., Birmingham, AL, USA; goat anti-mouse IgG2a peroxidase conjugates were from Abcam, Cambridge, UK. Trizol reagent was purchased from Invitrogen, Carlsbad, CA, USA; revert Aid™ M-MuLV reverse transcriptase was from Fermentas, USA; diethylpyrocarbonate (DEPC), ribonuclease inhibitor, and oligo(dT)_18_ were from Shanghai Sangon Biological Engineering Technology Co., Ltd., Shanghai, China; FastStart Universal SYBR Green Master (ROX) was from Roche Diagnostics Ltd., Shanghai, China. Agilent 4 × 44 k whole mouse genome microarray was provided from Agilent Technologies. Santa Clara, CA, USA.

### 2.2. Preparation and Characterization of AJSAF

AJSAF was prepared and characterized as previously described [15]. A total of 29 saponins including 10 new compounds in AJASF were identified and characterized by a high-performance liquid chromatography coupled with quadrupole time-of-flight mass spectrometry based on accurate mass database [17]. The endotoxin level in an AJSAF solution of 2 mg/mL was measured to be 0.253 ± 0.004 endotoxin units/mL by a tachypleus amebocyte lysate assay, indicating that AJSAF used in this study could be excluded from endotoxin contamination.

### 2.3. Mice

Female BALB/c mice aged 4–6 weeks were purchased from the Shanghai Experimental Animal Center, Chinese Academy of Sciences, Shanghai, China. Mice were acclimatized for one week prior to use. Rodent laboratory chow and tap water were provided ad libitum and maintained under controlled conditions with a temperature of 24 ± 1 °C and humidity of 50% ± 10%, and a 12/12 h light/dark cycle. All experiments were in compliance with the People’s Republic of China legislation on the use and care of laboratory animals, and followed the guidelines established by the Institute of Laboratory Animals of Zhejiang University, and approved by the University Animal Experimental Committee (no. 14878).

### 2.4. Immunization

To evaluate the adjuvant effects of AJSAF on rL-H5 vaccine, mice were divided into four groups, each consisting of five or 12 mice. Animals were immunized subcutaneously (*s.c.*) with rL-H5 (10^6^ EID_50_/dose) [18] alone or in combination with AJSAF (100 μg) at the same leg (AJSAF+rL-H5) or at different legs (AJSAF/rL-H5) on Day 1. A boosting injection was given two weeks later. Animals injected with 200 μL of PBS were included as a negative control. Sera and splenocytes were collected two weeks after the second immunization for measurement of the H5Ag-specifc antibody, gene microarray, and quantitative real-time PCR (qRT-PCR).

For 2D DIGE–MALDI-TOF-MS, mice were divided into two groups, each consisting of 12 mice. Animals were immunized *s.c.* with rL-H5 alone or in combination with AJSAF (100 μg) at the same leg (AJSAF+rL-H5) on Day 1. A boosting injection was given two weeks later. Splenocytes were collected two weeks after the second immunization.

### 2.5. Measurement of H5Ag-Specific Antibody

The serum H5Ag-specific IgG, IgG1, IgG2a, and IgG2b antibodies were detected in individual serum samples by an indirect ELISA as previously described [18]. The optical density was measured in a BIO-RAD 680 ELISA reader at 492 nm, where sets of sera samples have been subjected within and between group comparisons, and ELISA assays were performed on the same day for all of the samples.

### 2.6. Quantitative Real-Time PCR (qRT-PCR)

Splenocytes from immunized mice were incubated with H5Ag (final concentration 0.125 hemagglutinating units (HAU)/ml) for 12 h. The total RNA was isolated with TRIzol reagent and reverse transcription was performed as previously [19]. The PCR was performed with FastStart universal SYBR Green Master (ROX) on a BioRad CFX96 system. Primers for qRT-PCR were synthesized by Shanghai Sangon Biological Engineering Technology Co., Ltd., China, and the sequences were listed in Supplementary Table S1. The qPCR cycling was performed as follows: Initial denaturation at 95 °C for 10 min followed by 40 cycles of denaturation at 95 °C for 10 s, and annealing for 60 s. GAPDH was used as an endogenous control. The mRNA expression levels of the tested genes relative to GAPDH were determined using the 2^−ΔΔCt^ method and as fold induction [19].

### 2.7. Microarray Analysis

The splenocytes were stimulated with H5Ag (final concentration 0.125 HAU/ml) for 12 h. Splenocytes from four mice per group were pooled and total RNA was extracted using a TRIzol reagent and further purified with the RNeasy® Mini kit (Qiagen). RNA quality was assessed by the 2100 Bioanalyzer automated microfluidic system in combination with the RNA 6000 Nano kit (Agilent) following the producer’s protocol. Agilent 4 × 44 k whole mouse genome microarray representing 41,174 probes was used, and the RNA labelling and microarray hybridization were carried out according to the Agilent one-color microarray-based gene expression analysis protocol. Hybridized microarrays were scanned with a DNA microarray scanner (G2565BA, Agilent Technologies) and features were extracted using the Feature Extraction software 10.7 (Agilent Technologies) using default protocols and settings (scan resolution = 5 μm, PMT 100%). Data pre-processing and differential expression analysis of the gene expression data were done in R (v2.10.0, Bioconductor). Data were normalized between arrays using the quantile method (GENESPRING12.0). Normalized expression data was subjected to log2 transformation. Significantly regulated probes were selected by the following cutoff: Fold change (FC) > 2 and *p* < 0.05. Gene set enrichment analysis (GSEA) on “adaptive immune response (GO: 0002250)” was performed using the analysis software provided by the Broad Institute (Cambridge, MA) [20]. Venn diagram was performed in R with Vennerable package (v3.5.1). The common genes of AJSAF+rL-H5 and AJSAF/rL-H5 were defined as differentially expressed genes (DEGs) in both groups but with no significantly different expression; AJSAF+rL-H5-specific genes were defined as the DEGs in AJSAF+rL-H5 group with significantly different expression compared to AJSAF/rL-H5, while AJSAF/rL-H5-specific genes were defined as DEGs in AJSAF/rL-H5 group with significantly different expression compared to AJSAF+rL-H5. The pathway and function enrichment analysis of each gene set was performed using Metascape (http://metascape.org/gp/index.html#/main/step1) [21]. The network analysis of protein-protein interactions (PPI) within each gene set was carried out based on IMEx Interactome by InnateDB (http://www.innatedb.com/) [22] and visualized using Cytoscape (v3.6.0) [23].

### 2.8. Two-Dimensional Difference Gel Electrophoresis (2D DIGE)

The splenocytes were stimulated with H5Ag (final concentration 0.125 HAU/ml) for 24 h. Splenocytes from four mice per group were pooled and solubilized in the DIGE lysis buffer by ultrasound on ice (80 W, 10 s each time, 5 times, 15 s apart). The supernatant was collected by centrifugation at 12,000× *g* for 45 min and the protein contents were determined using the Protein Assay kit (Bio-Rad). For two-dimensional gel electrophoresis (2-DE), 100 μg and 1 mg of proteins were loaded onto analytical and preparative gels, respectively. The Ettan IPGphor Isoelectric Focusing System (GE Amersham) and pH 3-10 immobilized pH gradient (IPG) strips (13 cm, nonlinear; GE Healthcare) were used for isoelectric focusing (IEF). The IPG strips were rehydrated for 12 h in 250 μL of rehydration buffer containing the protein samples. IEF was performed in four steps: 30 V for 12 h, 500 V for 1 h, 1000 V for 1 h, and 8000 V for 8 h. The gel strips were equilibrated for 15 min in an equilibration buffer (50 mM Tris-HCl (pH 8.8), 6 M urea, 2% SDS, 30% glycerol, and 1% DTT). This step was repeated using the same buffer with 4% iodoacetamide in place of 1% DTT. The strips were then subjected to the second-dimensional electrophoresis after transfer onto 12.5% SDS-polyacrylamide gels. Electrophoresis was performed using the Hofer SE 600 system (GE Amersham) at 15 mA per gel for 30 min, followed by 30 mA per gel until the bromophenol blue reached the end of the gel. Three replicates were performed for each sample. Protein spots in the nine gels were visualized by silver staining to estimate the 2-DE feasibility of all samples.

For 2D DIGE, 50 μg proteins per sample was labeled with 400 pmol of Cy3 or Cy5 fluorescent dyes (GE Healthcare) and 50 μg of equal mixture from three samples was labeled with 400 pmol of Cy2 fluorescent dye (GE Healthcare) as an internal standard. The labeling reaction was carried out in the dark on ice for 30 min, and quenched with 10 mM lysine for 10 min. The Cy2, Cy3, and Cy5-labeled samples were mixed and 2-DE was performed as described above. The images were acquired on a UMax Powerlook 2110XL (GE Healthcare) at the excitation/emission of 488/520, 532/580, and 633/670 nm, respectively, and analyzed with the DeCyder Image-Quant™ software (GE Healthcare). Protein spots were represented in all gels (*n* = 3) with expression level greater than 1.2-FC and *p* < 0.05 were defined as being differentially expressed and selected for further characterization.

### 2.9. Protein Identification by MALDI-TOF-MS

All the differentially expressed spots were selected and excised manually from the preparative gels. Protein spots of interest were cut from the preparative gels, destained for 20 min in 30 mM potassium ferricyanide/100 mM sodium thiosulfate (1:1 v/v), and washed with Milli-Q water until the gels were destained. The spots were incubated in 0.2 M NH_4_HCO_3_ for 20 min and then lyophilized. Each spot was digested overnight in 12.5 ng/µL trypsin in 25 mM NH_4_HCO_3_. The peptides were extracted three times with 60% acetonitrile (ACN)/0.1% trifluoroacetic acid (TFA). The extracts were pooled and dried completely by a vacuum centrifuge.

MS and MS/MS data for protein identification were obtained by using a MALDI-TOF-TOF instrument (5800 proteomics analyzer; Applied Biosystems). Instrument parameters were set using the 4000 Series Explorer software (Applied Biosystems). The MS spectra were recorded in the reflector mode in a mass range from 800 to 4000 with a focus mass of 2000. The TOF/TOF calibration mixtures (AB SCIEX) were used to calibrate the spectrum to a mass tolerance within 10 ppm. The MS spectra were processed using the TOF-TOF Series Explorer software (v4.0, AB SCIEX). At least 1000 laser shots were typically accumulated with a laser pulse rate of 400 Hz in the MS mode, whereas in the MS/MS mode spectra up to 2000 laser shots were acquired and averaged with a pulse rate of 1000 Hz. For MS calibration, autolysis peaks of trypsin ([M+H]^+^ 842.5100 and 2211.1046) were used as internal calibrates, and the most intense ion signals (up to 10) were selected as precursors for MS/MS acquisition, excluding the trypsin autolysis peaks and the matrix ion signals.

The peptide mass finger printing (PMF) and MS/MS queries were performed using the MASCOT search engine 2.2 (Matrix Science, London, UK) embedded into the GPS-Explorer Software 3.6 (Applied Biosystems) on the NCBI protein database with the following parameter settings: Mass accuracy 100 ppm, trypsin cleavage one missed cleavage allowed, carbamidomethylation set as fixed modification, oxidation of methionine was allowed as variable modification, and MS/MS fragment tolerance was set to 0.4 Da. A GPS Explorer protein confidence index ≥ 95% were used for further manual validation.

### 2.10. Statistical Analysis

The normality of the distribution of each variable was measured through means of the Kolmogorov–Smirnov test. Data were expressed as mean ± SEM and examined for their statistical significance of difference with ANOVA and a Tukey post-hoc test. The calculations and graphs were produced using the Prism 7 software (GraphPad Software, San Diego, CA, USA). A *p*-value less than 0.05 were considered to be statistically significant.

## 3. Results

### 3.1. Comparative Analysis of H5Ag-Specific Serum Antibody Response

**Figure 1:**
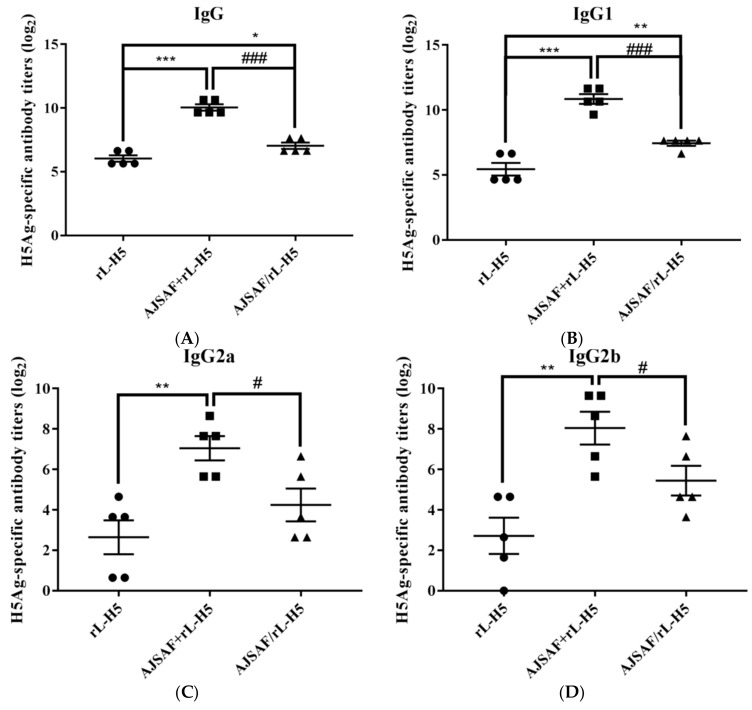
Serum H5Ag-specific IgG (**A**), IgG1 (**B**), IgG2a (**C**), and IgG2b (**D**) antibody titers in mice immunized with Newcastle disease virus-based recombinant influenza vaccine alone (rL-H5) or in combination with the purified active fraction of *Albizia julibrissin* saponin (AJSAF) at the same leg (AJSAF+rL-H5) or different legs (AJSAF/rL-H5). The values are presented as mean ± SEM (*n* = 5). *P*-values were determined by ANOVA and a Tukey post-hoc test. Significant differences with the rL-H5 alone group were designated as * *p* < 0.05, ** *p* < 0.01, and *** *p* < 0.001; those with the AJSAF/rL-H5 group as ^#^ *p* < 0.05, ^##^ *p* < 0.01, and ^###^ *p* < 0.001.

The serum H5Ag-specific IgG, IgG1, IgG2a, and IgG2b antibody levels were measured two weeks after the last immunization using ELISA, and the results were shown in Figure 1. The rL-H5 alone induced the low serum H5Ag-specific IgG and its isotypes antibody liters. The addition of AJSAF to rL-H5 (AJSAF+rL-H5) resulted in a significant increase in serum H5Ag-specific IgG, IgG1, IgG2a, and IgG2b antibody titers (*p* < 0.01 or *p* < 0.001). However, the immunization by injection of AJSAF and rL-H5 in mouse different legs (AJSAF/rL-H5) only significantly enhanced the serum H5Ag-specific IgG and IgG1 titers in the rL-H5-immunized mice compared with the rL-H5 alone group (*p* < 0.05 or *p* < 0.01) (Figure 1A,B). There were, however, no significant differences in the serum H5Ag-specific IgG2a and IgG2b titers between rL-H5 alone and AJSAF/rL-H5 groups (*p* > 0.05) (Figure 1C,D). Moreover, the serum H5Ag-specific IgG, IgG1, IgG2a, and IgG2b antibody titers in AJSAF/rL-H5-immunized mice were significantly lower than those in the AJSAF+rL-H5 group (*p* < 0.05, or *p* < 0.001). The antigen-specific IgG2a/2b and IgG1 are markers for Th1 and Th2 responses, respectively. Therefore, the adjuvant activity of AJSAF on Th1 and Th2 responses was dependent on the colocalization of AJSAF and antigen.

### 3.2. Comparative Analysis of Global Gene Expression

**Figure 2:**
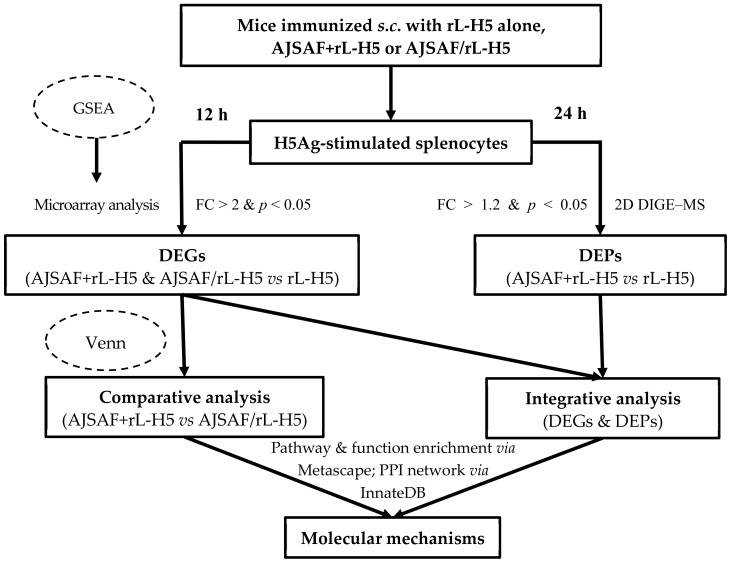
Workflow of the transcriptomic and proteomic analyses.

**Figure 3:**
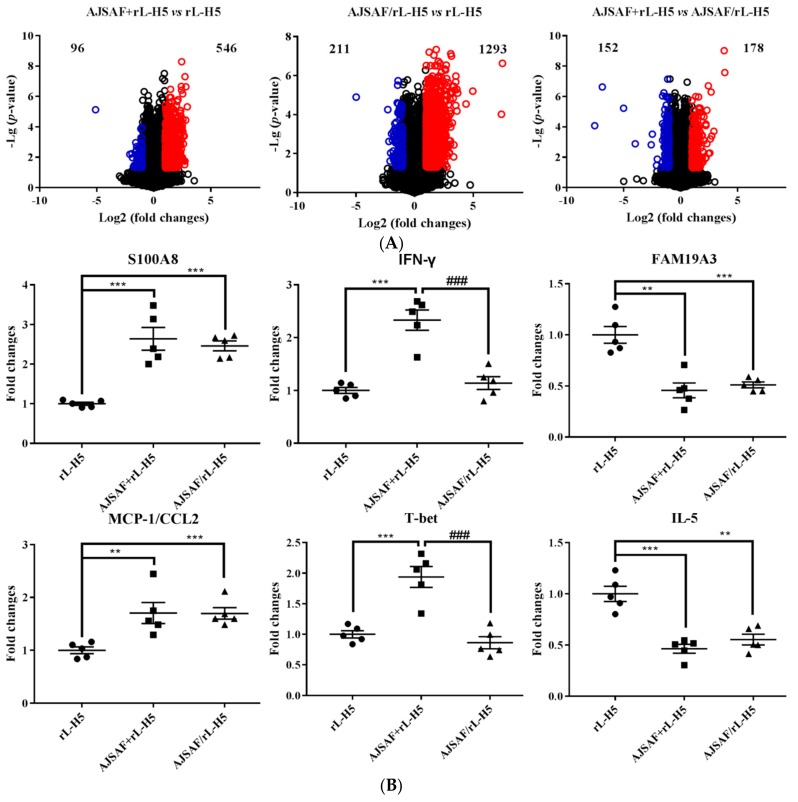

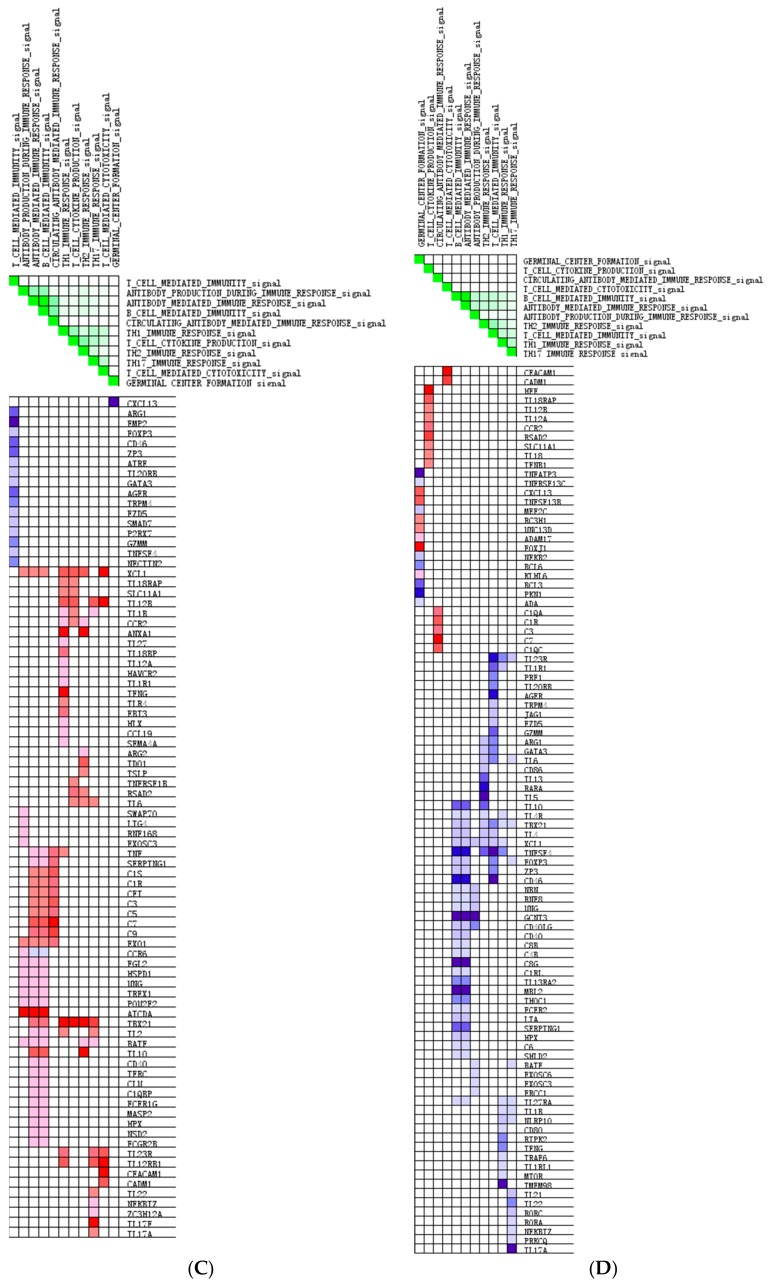
Expression profiles of differentially expressed genes (DEGs) in H5Ag-stimulated splenocytes from the mice immunized with rL-H5 alone (rL-H5) or in combination with AJSAF at the same leg (AJSAF+rL-H5) or different legs (AJSAF/rL-H5). (**A**) Volcano plots. (**B**) qRT-PCR verification. *P*-values were determined by ANOVA and a Tukey post-hoc test. The values are presented as mean ± SEM (*n* = 5). Significant differences with rL-H5 alone group were designated as * *p* < 0.05, ** *p* < 0.01, and *** *p* < 0.001; those with AJSAF/rL-H5 group as ^#^ *p* < 0.05, ^##^ *p* < 0.01, and ^###^ *p* < 0.001. (**C**,**D**) Heatmap of enriched DEGs on “adaptive immune response (GO: 0002250)” in AJSAF+rL-H5 (**C**) and AJSAF/rL-H5 (**D**) compared to rL-H5 alone using gene set enrichment analysis (GSEA).

To gain insight into the molecular mechanisms underlying the responses to the different regimens of AJSAF and rL-H5, gene microarray and 2D DIGE–MALDI-TOF-MS were used to analyze the transcriptomic and proteomic profiles of H5Ag-stimulated splenocytes from immunized mice (Figure 2). The splenocytes stimulated with H5Ag for 12 h were subjected to microarray analysis. Six hundred and forty-two differentially expressed probes were identified in AJSAF+rL-H5 group relative to rL-H5 alone group with FC > 2 and *p* < 0.05 calculated on the three replicates (Figure 3A, right). Among them, 546 were upregulated and 96 were downregulated, corresponding to 411 and 66 genes, respectively, after correcting for redundant probes and excluding unknown genes. Unexpectedly, a larger number of DEGs were identified for AJSAF/rL-H5 group. There were 1504 differentially expressed probes in AJSAF/rL-H5 group compared to rL-H5 alone group. Among them, 1293 were upregulated and 211 were downregulated, corresponding to 917 and 169 genes, respectively (Figure 3A, middle). The comparison of AJSAF+rL-H5 group with AJSAF/rL-H5 group revealed 330 differentially expressed probes, covering 178 upregulated and 152 downregulated probes, corresponding to 145 and 121 genes, respectively (Figure 3A, left). To confirm the validity of the microarray data, qRT-PCR was undertaken for putative six DEGs (S100A8, MCP-1/CCL2, IFN-γ, T-bet, FAM19A3, and IL-5), and the results were shown in Figure 3B and Figure S1. The qRT-PCR results were consistent with the microarray data except for IFN-γ. In qRT-PCR results, the mRNA expression levels of IFN-γ in AJSAF/rL-H5 group were significantly lower than those in AJSAF+rL-H5 group (*p* < 0.001). There was, however, no significant difference between rL-H5 alone and AJSAF/rL-H5 groups (*p* < 0.05). In microarray data, the mRNA expression levels of IFN-γ in AJSAF/rL-H5 group were significantly lower than those in both rL-H5 alone and AJSAF+rL-H5 groups for two designed IFN-γ probes (*p* < 0.001).

GSEA of DEGs in AJSAF+rL-H5 and AJSAF/rL-H5 groups compared to rL-H5 alone group was conducted to compare the statistically significant differences of gene expression in the defined gene sets across “adaptive immune response (GO: 0002250)”. There was, however, no difference in the enriched gene sets in both AJSAF+rL-H5 and AJSAF/rL-H5 groups compared to rL-H5 alone group (FDR > 25%, Figure S2). Therefore, an alternative was used to focus on enriched individual genes to identify functional molecular signatures (Figure 3C,D). Compared to rL-H5 alone group, CEACAM1 and CADM1 for “circulating antibody mediated immune response”, as well as C3, C7, and C1RA/B for “T cell mediated cytotoxicity” were enriched in AJSAF+rL-H5 and AJSAF/rL-H5 groups. Compared to AJSAF/rL-H5 groups, the more upregulated genes involved in adaptive immunity, such as IFN-γ, T-bet (TBX21), ANXA1, IL-12RB1, TNF, and IL-12β for “Th1 immune response”, IL-10, RSAD2, and IL-6 for “Th2 immune response”, IL-17F for “Th17 immune response”, and AICDA, XCL1, and IL-10 for “B cell mediated immunity”, were enriched in AJSAF+rL-H5 group. Notably, T-bet was identified as a core enriched gene for Th1, Th2, and Th17 responses. T-bet is a core transcription factor promoting Th1 response [24] and negatively regulating Th2 and Th17 responses [25,26]. In addition, it was found that the heterogeneity of splenocytes could limit the sensitivity of gene expression such as IL-12β, IL-6, and TNF (1.5 < FC < 2 and *p* < 0.05).

### 3.3. Pathway and Function Analysis of DEGs

**Figure 4:**
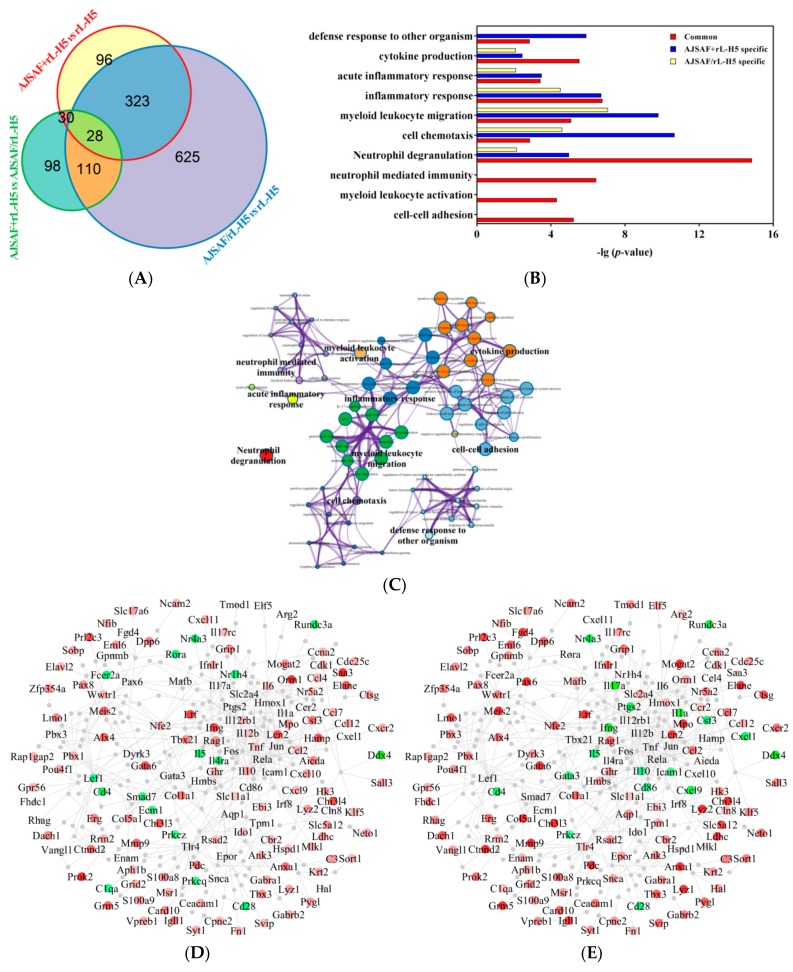

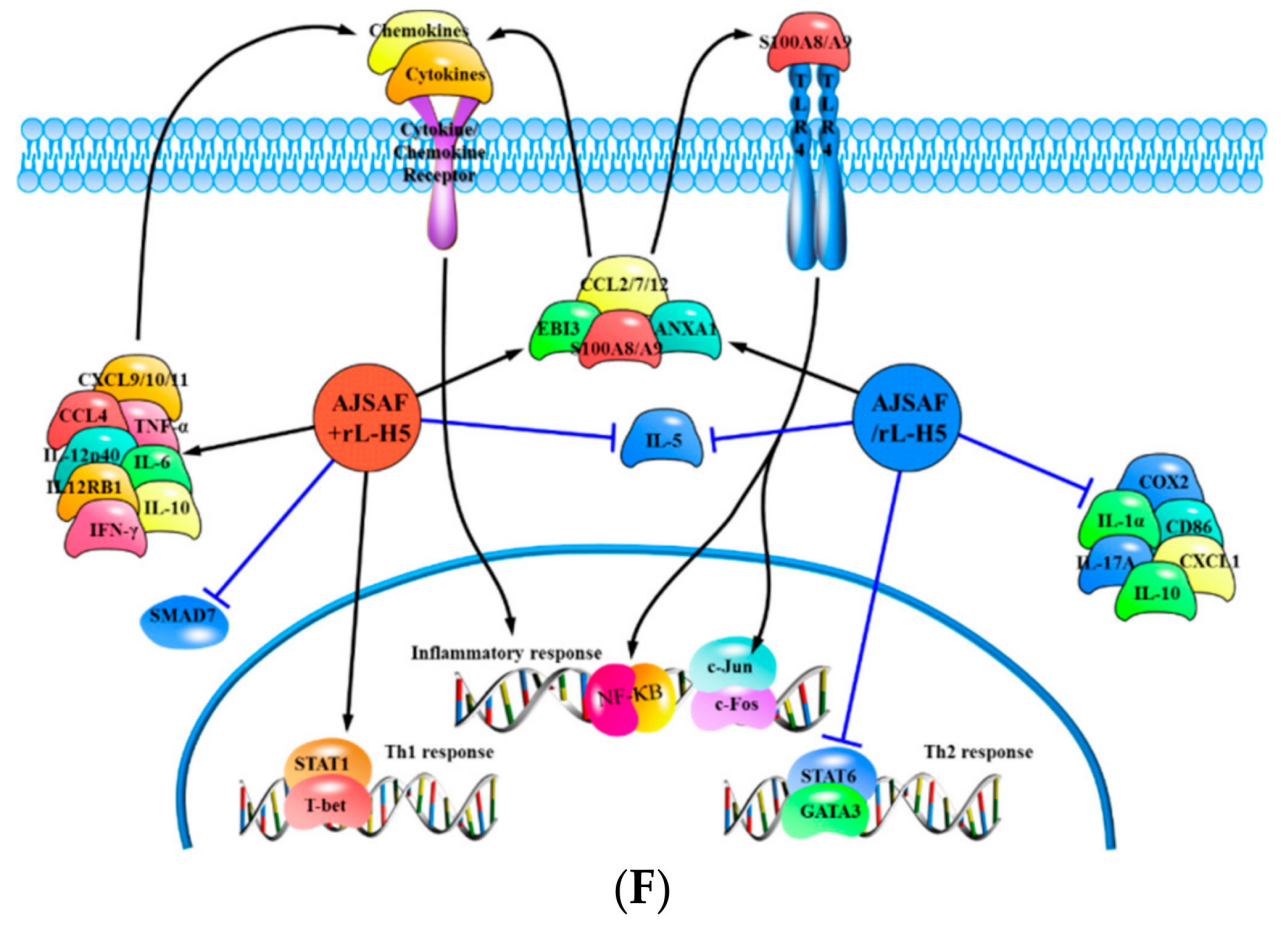
Function and pathway of differentially expressed genes (DEGs) in H5Ag-stimulated splenocytes from the mice immunized with rL-H5 alone (rL-H5) or in combination with AJSAF at the same leg (AJSAF+rL-H5) or different legs (AJSAF/rL-H5). (**A**) Venn diagram. (**B**,**C**) Enriched function and pathway (**B**) and network visualization (**C**) of DEGs. (**D**,**E**) Protein-protein interaction (PPI) network of DEGs in AJSAF+rL-H5 (**D**) and AJSAF/rL-H5 (**E**) compared to rL-H5 alone with upregulated and downregulated DEGs expressed in red and green, respectively. (**F**) Proposed mechanisms of adjuvant activity of AJSAF based on the transcriptomic profile.

In view of that the colocalization of AJSAF with antigen was required for its adjuvant activity, to identify the gene signatures mediated its adjuvant activity, DEGs were classified into three categories: AJSAF+rL-H5-specific, AJSAF/rL-H5-specific, and their common genes, including 58, 138, and 323 DEGs, respectively (Figure 4A). Among AJSAF+rL-H5-specific DEGs, IFN-γ, IL-12Rβ1, IL-10, T-bet, and AICDA were related to “adaptive immune response”.

Next, the function enrichment analysis of DEGs was performed, and the results were shown in Figure 4B. The common DEGs were specifically enriched in “cell-cell adhesion”, “myeloid leukocyte activation”, and “neutrophil mediated immunity”. These common DEGs were also involved in “Neutrophil degranulation” with a higher *p*-value (*p* = 1.438E-15). AJSAF+rL-H5-specific DEGs were enriched in “cell chemotaxis”, “myeloid leukocyte migration”, and “defense response to other organism” with a higher *p*-value than the other two categories. There were, however, no specific terms enriched in AJSAF/rL-H5-specific DEGs. The relationships of these enriched terms were visualized as an integrated network in Figure 4C. It revealed “myeloid leukocyte activation” as a bridge connected “neutrophil mediated immunity” and other enriched terms. Meanwhile, “neutrophil mediated immunity” also directly connected “cell-cell adhesion” and “myeloid leukocyte migration” by the inter-cluster similarities. The PPI network analysis of the three gene groups established a biological network, consisting of 322 nodes and 584 edges (Figure 4D,E). It contained 78 common genes, most of which (e.g., ANXA1, C3, CCL12, CCL2, CEACAM1, CTSG, CXCR2, ELANE, GATA6, MMP9, NFIB, S100A8, and S100A9) were significantly upregulated. Compared to rL-H5 alone group, 22 genes including AICDA, CCL4, CSF3, CXCL10, CXCL11, IFN-γ, IL-10, IL-12RB1, SAA3, and T-bet were specifically upregulated in AJSAF+rL-H5 group, but not significantly changed or even downregulated in AJSAF/rL-H5 group. The mRNA expression of CXCL1, CXCL9, IL-17α, IL-1α, and PTGS2 were specifically downregulated in AJSAF/rL-H5 group compared to rL-H5 alone group. IRF8, RELA, and JUN were identified as central hubs of the network. For AJSAF+rL-H5 group, a cluster of upregulated genes including AICDA, CHI3l4, CXCL9, HK3, IDO1, IL-12β, LDHC, LYZ2, MLKL, SLC11A1, SLC5A12, and TNF was connected by IRF8. Both RELA and JUN could upregulate the mRNA expression of CCL2, CXCL10, IL-10, IL-12β, IL-6, and TNF. In addition, RELA specifically regulated the mRNA expression of CSF3, HAMP, IDO1, LCN2, RAG1, and T-bet, while JUN specifically upregulated the mRNA expression of CCL12, CCL4, CCL7, CXCL9, and IFN-γ.

The molecular mechanisms of AJSAF colocalized with antigen or not were compared *via* integrating the microarray data (Figure 4F). The upregulated S100A8 and S100A9 in both AJSAF+rL-H5 and AJSAF/rL-H5 groups could be recognized by TLR4, leading to activation of NF-κB (RELA) and AP-1 (JUN and FOS) and induction of Th1 response. Th1-related genes (e.g., ANXA1, EBI3, CCL2, CCL7, and CCL12) and Th2-related genes (e.g., RSAD2) were upregulated, and IL-5, a typical gene of Th2 response, was downregulated in both AJSAF+rL-H5 and AJSAF/rL-H5 groups. In AJS+rL-H5 group, Th1 (e.g., T-bet, IFN-γ, TNF-α, IL-12β, IL12Rβ1, CCL4, CXCL9, CXCL10, and CXCL11), Th2 (IDO1, EDN1, IL-6, and IL-10), and Th17 (IL-6, IL-12β, and IL12Rβ1) immune response genes (IRGs) were upregulated, and SMAD7, a negative regulator for Th17 response, was downregulated. In AJSAF/rL-H5 group, several IRGs including GATA3, IL-1α, PTGS2, CD86, IL-17α, CXCL1, and IL-10 were downregulated.

### 3.4. Integrative Analysis of Transcriptomic and Proteomic Profiles

**Figure 5:**
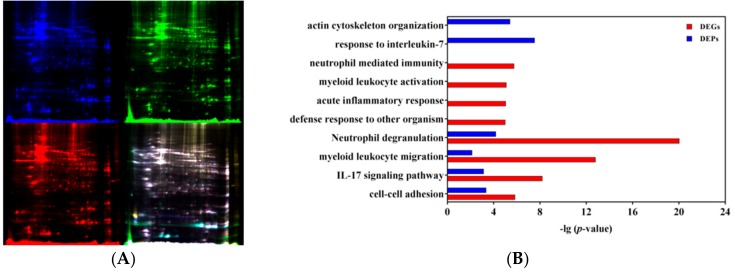

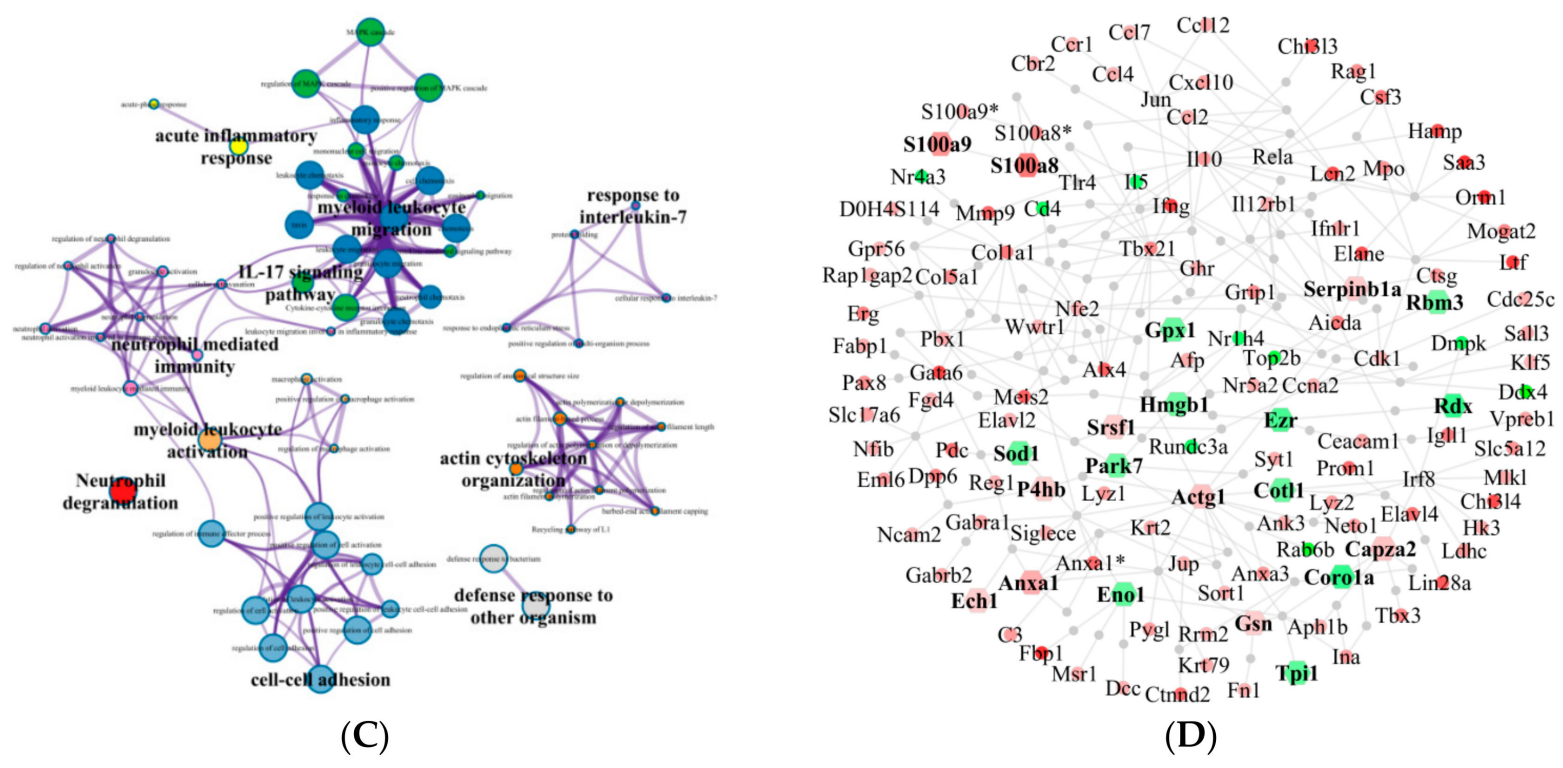
Function and pathway of differentially expressed proteins (DEPs) in H5Ag-stimulated splenocytes from the mice immunized with rL-H5 alone (rL-H5) or in combination with AJSAF at the same leg (AJSAF+rL-H5). (**A**) Two-dimensional difference gel electrophoresis (2D DIGE) of proteins. (**B**,**C**) Enriched function and pathway (**B**) and network visualization (**C**) of DEGs and DEPs. (**D**) Protein-protein interaction (PPI) network of DEGs (circle) and DEPs (hexagon) with upregulated and downregulated in red and green, respectively.

The proteomic analysis of H5Ag-stimulated splenocytes from the mice immunized with rL-H5 was performed using 2D DIGE–MALDI-TOF-MS (Figure 5A). Among 1637 detected protein spots, 52 differentially expressed protein (DEP) spots were found in AJSAF+rL-H5 group compared with rL-H5 alone group with FC > 1.2 and *p* < 0.05 calculated on the three replicates. Among the DEP spots, a total of 48 spots were identified, including 23 upregulated and 25 downregulated, corresponding to 18 and 23 proteins, respectively (Table 1).

To compare the transcriptomic and proteomic profiles, 477 DEGs and 41 DEPs in AJSAF+rL-H5 group compared with rL-H5 alone group were used. It revealed only five in common, including NGP, S100A8, S100A9, ANXA1, and CAMP. The limited consistence of transcriptomic and proteomic data might result from the marked spatial, temporal, and quantitative differences between mRNA and protein expression [27]. On the other hand, it was also restricted by some technical factors such as detection depth difference, screening standard for differential expression, and the limited detection time points. Thus, it highlights the integrated analysis of transcriptomic and proteomic profiles [28]. Beyond single-gene-level analysis, the expression of pathways can be also evaluated directly, which provides a more specific biological context and increasing statistical power [29]. The pathway and function enrichment analysis of DEGs and DEPs was performed, and the comparative results of enriched terms were shown in Figure 5B. Both DEGs and DEPs were significantly enriched in “cell-cell adhesion”, “IL-17 signaling pathway”, “myeloid leukocyte migration”, and “Neutrophil degranulation” (*p* < 0.001). The terms “defense response to other organism”, “acute inflammatory response”, “myeloid leukocyte activation”, and “neutrophil activation” were specifically enriched for DEGs, and “response to interleukin-7” and “actin cytoskeleton organization” for DEPs (*p* < 0.001). The same and different enrichment results between DEGs and DEPs suggested the temporal order and duration of processes induced by AJSAF. As the enrichment network visualization shown in Figure 5C, the terms “response to interleukin-7” and “actin cytoskeleton organization” specifically enriched for DEPs were independent without connections with other enriched terms based on the latest databases. By setting DEPs as primary nodes and DEGs as the second, the PPI network of 226 nodes and 342 edges were established (Figure 5D). It included 10 upregulated (ACTG1, ANXA1, CAPZA2, ECH1, GSN, P4HB, S100A8, S100A9, SERPINB1A, and SRSF1) and 11 downregulated (CORO1A, COTL1, ENO1, EZR, GPX1, HMGB1, PARK7, RBM3, RDX, SOD1, and TPI1) DEPs. It revealed that S100A8/A9 was differentially expressed in both gene and protein levels, and connected to TLR4, a critical PPR for Th1 response. Most DEPs indirectly connected with DEGs, but SERPINB1A directly connected with AICDA, CTSG, and ELANE.

## 4. Discussion

AJSAF is an ideal adjuvant candidate that induces antigen-specific of both cellular and humoral immune responses with mixed Th1/Th2 responses [14,15]. AJSAF has been recently reported to activate RAW264.7 cells via Ca^2+^–ERK1/2–CREB pathways [30]. However, its in vivo mechanisms of adjuvant activity have not been well clarified yet. Moreover, the mechanisms of Th1 or Th2 selectivity of adjuvants remain inconclusive, resulting in a shortage of guidelines for designing selective adjuvants. In this study, the mechanisms of action of AJSAF were explored by comparing adaptive immune response in mice immunized with rL-H5 and AJSAF at the same leg or different legs. It revealed that AJSAF-mediated antibody response was dependent on spatial colocalization of AJSAF and antigen.

AJSAF+rL-H5 specifically upregulated several typical Th1 IRGs including T-bet, IFN-γ, TNF-α, IL-12β, and IL-12Rβ1 (Figure 4F). Th1 response was also characterized by the common genes (e.g., S100A8, S100A9, ANXA1, and EBI3) and functions (e.g., “inflammatory response” and “neutrophil mediated immunity”). The upregulated mRNA expression of S100A8, S100A9, and ANXA1 in AJSAF+rL-H5 group was also verified by proteomic data. S100A8 and S100A9 are calcium- and zinc-binding proteins and involve in the regulation of inflammatory processes and immune response. During inflammation, S100A8 and S100A9 regulate the inflammatory response characterized by leukocyte recruitment and cytokine secretion through activating RAGE and TLR4 [31]. S100A8 and S100A9 could induce DC maturation [32] and contribute to the development of autoreactive CD8^+^ effectors through TLR4 [33]. Recently, S100A8 and S100A9 have been defined as alarmins in driving adaptive immune responses. Alarmins are a subset of endogenous DAMPs that interact with PRRs such as TLRs as cytokine-like mediators that participate in host defense and are usually not dangerous [34]. Alarmins are distinct from other endogenous DAMPs in their efficacy as endogenous immunoenhancing adjuvants [35,36]. S100A8 and S100A9 were reported to be Th1-polarizing alarmins to shape the types of adaptive immune response [34]. Therefore, AJSAF-induced predominant elevation of S100A8 and S100A9 was proposed to be required for its adjuvanticity. Another Th1-polarizing alarmin, high-mobility group box 1 protein (HMGB1), has also been demonstrated to exhibit adjuvant activity [37]. However, HMGB1 was significantly downregulated in AJSAF+rL-H5 group, thus suggesting its distinct role. More studies are warranted to clarify the role of alarmins in AJSAF-mediated immune responses.

The “inflammatory response” is another common property, independent of colocalization of AJSAF and rL-H5. The role of inflammation in adjuvant activity remains inconclusive. However, it is undoubted and unarguable that the overly aggressive or prolonged inflammation affects the safety of adjuvants. Annexin A1 (ANXA1) is an anti-inflammatory mediator with pro-resolving properties. ANXA1 has been shown to exert various anti-inflammatory actions: (i) Inhibited neutrophil recruitment, (ii) induced neutrophil apoptosis, (iii) promoted monocyte recruitment, (iv) activated the clearance of apoptotic leukocytes by macrophages, and (v) yielded macrophage reprogramming from a pro-inflammatory to a pro-resolving phenotype [38]. Evidence indicated that the externalization of ANXA1 and then its interaction with the formyl peptide receptor type 2/lipoxin A4 receptor (FPR2/ALX) were required for its anti-inflammatory effects [39,40,41]. The microarray data revealed that AJSAF significantly upregulated the mRNA expression of ANXA1 and FPR2 in mice immunized with rL-H5 suggesting the involvement of ANXA1-FPR2/ALX signaling in the anti-inflammatory effects of AJSAF. The upregulation of neutrophil elastases (ELANE, CTSG, and PRTN3) and their fast-acting inhibitor SERPINB1A indicated another anti-inflammatory mechanism of AJSAF [42]. Accompanied by the initiation of acute inflammation, AJSAF induced effective anti-inflammatory response to prevent the excessive and prolonged inflammation, reducing its side effects.

Unlike Th1 response, there were limited numbers of classical Th2 genes upregulated in AJSAF+rL-H5 group, and it failed to discover the precise signaling events or crucial factors to interpret the mechanisms of action of AJSAF-mediated Th2 responses. IL-10 helps to polarize Th2 immune response by preferentially suppressing the production of IL-12 and IFN-γ from Th1 cells [43]. However, Khan et al. [44] reported that the endogenous IL-10 is not a switch factor for IgG1. Thus, except IL-10, other mediators might contribute to Th2-polarized antibodies induced by AJSAF. Accumulating evidence suggests that most adjuvants trigger early innate immune responses to induce robust and long-lasting adaptive immune responses [45]. The observation that AJSAF-mediated Th2 response was dependent on the colocalization of AJSAF with antigen suggested a causative role of the local innate immune response triggered by AJSAF. In our previous study, adjuvant-active fraction from *A. julibrissin* saponin induced the production of Th2 cytokines (IL-13 and IL-9) at the injection site [14]. Whether the local innate immune response triggered by AJSAF regulates subsequent Th2 responses is being elucidated.

In addition, “actin cytoskeleton organization” might mediate Th1 and Th2 immune responses induced by AJSAF. Actin, a major component of the cytoskeleton, is a dynamic polymer. Its monomer is globular (G-actin, encoded by ACTG1) and forms various shaped filaments (F-actin) when polymerized. The dynamic nature of actin polymerization and depolymerization is central to functions of actin network, which is regulated by numerous actin binding proteins (ABPs). Recently, the importance of cytoskeletal function in immunity has been well-recognized. Beyond enabling cell migration and adhesion, the actin network is essential for many facets of innate and adaptive immunity, including phagocytosis, leukocyte activation, and immune synapse formation [46]. Gelsolin (GSN), one of the most abundant ABPs, regulates actin by severing, capping, nucleating actin filaments, and sequestering monomers [47]. It was reported that GSN participated in immunological processes such as phagocytosis [48], macrophage recruitment, and motility [49], as well as neutrophil regulation and adhesion [50]. Capping actin protein of muscle Z-line subunit alpha 2 (CAPZ) binds to the fast-growing barbed ends of actin filaments thereby blocking the exchange of subunits at these ends. Coactosin-like protein 1 (COTL1), a member of the actin-depolymerizing factor (ADF)/cofilin family, was shown to bind F-actin, but not G-actin [51]. COTL1 competes with cofilin for binding to F-actin, and then attenuates cofilin-mediated F-actin depolymerization to promote lamellipodial protrusion [52]. Coronin 1A (CORO1A) is a member of the coronin family that function as important regulators of the actin cytoskeleton. It regulated the innate and adaptive immune responses in an actin-dependent manner [53], such as enhancing neutrophil phagocytosis [54], and regulating chemokine-mediated T-cell migration [55]. Ezrin (EZR), radixin (RDX) and moesin (MSN) belong to the ezrin-radixin-mesin (ERM) family of membrane-actin cytoskeleton crosslinkers and participate in a variety of cellular processes. These ABPs control the shape, cytokinesis, adhesion, and activation of T cell [56] and participate in immune synapse formation, an essential process for APC-T cell interaction [57]. It was reported that the size of B cell receptor (BCR) microclusters, and magnitude of BCR signaling and antigen-specific antibody production are increased in the absence of EZR [58] and that the conditional deletion of EZR in B cells increases IL-10 production induced by TLR4 ligation [59]. Although the protein level changes of G-actin and these ABPs were observed in our data, their functions in mediating the adaptive immune response remain to be elucidated.

## 5. Conclusions

**Figure 6:**
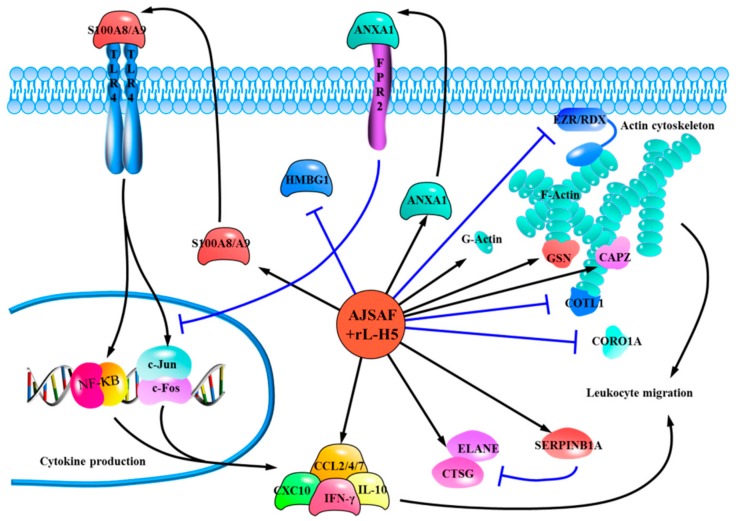
Proposed mechanisms of the adaptive immune response in mice induced by AJSAF based on the integrative analysis of transcriptomic and proteomic profiles.

The molecular mechanisms of action of AJSAF were comprehensively analyzed based on transcriptomic and proteomic profiles from the single gene level to the pathway, function, and network levels (Figure 6). In addition to the activation of S100A8/A9-TLR4-NF-κB/AP-1 pathway and production of Th1/Th2 cytokines, AJSAF was proposed to regulate: (i) The actin cytoskeleton, characterized by upregulation of ACTG1, CAPZ, and GSN, as well as downregulation of CORO1A, COTL1, EZR, and RDX; (ii) the leukocyte migration through affecting actin cytoskeleton and inducing chemokines (e.g., CCL2, CCL4, and CCL7); (iii) the anti-inflammatory response, including ANXA1-mediated anti-inflammatory effects via FPR2/ALX and SERPINB1A-mediated suppression of neutrophil elastases (ELANE and CTSG).

In this study, the mechanisms of action of Th1/Th2 responses induced by AJSAF were demonstrated based on the transcriptomic and proteomic profiles. The neutrophil response and its derived alarmin S100A8 and S100A9 might involve in the Th1 response. The dual nature of neutrophils is consistent with the benefit/risk profile of saponin-based adjuvants. Meanwhile, AJSAF might induce the adaptive immune responses by improving a local innate immune microenvironment. Our findings also highlight the important role of various alarmins in adjuvant studies due to their adjuvant efficacy, identified receptors, and downstream signal transducers. The insights obtained from this study further advance our understanding of the mechanisms of action of saponin-based adjuvants.

## Figures and Tables

**Table 1:** Differentially expressed protein spots successfully identified by matrix-assisted laser desorption/ionization time-of-flight mass spectrometry (MALDI-TOF-MS).

Spot No.	*p*-Value	Up/Down	Abbr	Accession No.	ID	MW	PI	Pep Count	Protein Score	Protein/Ion Credibility	Best Ion Score
L21	0.0031	3.38	NGP	IPI00127280	18054	19661.7	5.21	18	731	100/100	518
L24	0.00054	3.91	S100A8	IPI00230768	20201	10345.1	5.43	5	206	100/100	154
M1	0.0085	2.67				10345.1	5.43	3	279	100/100	252
M23	0.0017	3.05				10345.1	5.43	5	322	100/100	270
M24	0.017	3.02	S100A9	IPI00222556	20202	13211.3	6.64	9	383	100/100	311
N2	0.034	2.34				13211.3	6.64	10	426	100/100	339
L15	0.0032	2.31	ANXA1	IPI00230395	16952	38995.1	6.97	24	826	100/100	606
M11	0.019	1.92	EFHD2	IPI00112223	27984	26774.6	5.01	17	438	100/100	294
N8	0.00095	1.91	GM9234	IPI00987580	668548	18173.8	6.2	7	414	100/100	356
N9	0.00075	1.76				18173.8	6.2	8	526	100/100	456
L23	0.0096	1.8	CAMP	IPI00875797	12796	19797.3	8.9	3	159	100/100	142
M16	0.034	1.67	ACTG1	IPI01027491	11465	32941.3	5.15	6	107	100/100	75
M15	0.049	1.66	FTH1	IPI00230145	14319	21224.3	5.53	7	236	100/100	187
M6	0.037	1.63	ALDH2	IPI00111218	11669	57014.9	7.53	23	849	100/100	647
L14	0.013	1.56	CAPZA2	IPI00111265	12343	33117.7	5.57	11	347	100/100	260
M8	0.05	1.39	SERPINB1A	IPI00457659	66222	42718.8	5.85	22	1,130	100/100	928
L17	0.0012	1.38	PNP	IPI00607023	18950	32527.2	5.78	16	680	100/100	513
N20	0.0053	1.28	SRSF1	IPI00420807	110809	27841.9	10.37	9	82	99.96/93.89	35
M10	0.0066	1.27	GSN	IPI00759948	227753	80997.5	5.52	7	113	100/100	94
N19	0.042	1.25	ECH1	IPI00130804	51798	36437.3	7.6	14	603	100/100	493
M5	0.02	1.24	P4HB	IPI00133522	18453	57421.9	4.77	31	905	100/100	595
N16	0.044	1.2	PGAM1	IPI00457898	18648	28927.9	6.67	15	812	100/100	666
O3	0.022	1.25				28927.9	6.67	17	819	100/100	643
N14	0.0042	−2.12	BLVRB	IPI00113996	233016	22297.4	6.49	8	321	100/100	255
N13	0.0079	−1.83	IGK-C	IPI00850020	16071	24434.9	7.05	8	256	100/100	192
M4	0.03	−1.55	CORO1A	IPI00323600	12721	51641.2	6.05	15	447	100/100	339
M2	0.0017	−1.54	RDX	IPI00308324	19684	68614.4	5.91	15	247	100/100	183
M20	0.024	−1.48	SNRPF	IPI00943994	69878	9775.8	4.7	4	110	100/100	76
L11	0.0027	−1.45	EZR	IPI00330862	22350	69477.7	5.83	10	217	100/100	186
N12	0.019	−1.38	GUK1	IPI00986878	14923	22018.3	6.12	5	168	100/100	136
L22	0.0036	−1.38	RGS10	IPI00132450	67865	21194.6	6.36	15	500	100/100	347
N15	0.0065	−1.36	TPI1	IPI00988063	21991	27037.9	6.9	15	643	100/100	494
M7	0.0051	−1.33	ENO1	IPI00462072	13806	47453.3	6.37	21	628	100/100	431
N6	0.0013	−1.38	PPIA	IPI00554989	268373	18473.1	7.74	9	336	100/100	255
N7	0.0013	−1.27				18473.1	7.74	9	243	100/100	165
N18	0.0099	−1.3	EIF4H	IPI00124742	22384	27381.4	6.67	14	350	100/100	240
N3	0.0012	−1.28	SOD1	IPI00130589	20655	16103.9	6.02	13	645	100/100	500
N4	0.028	−1.3				16103.9	6.02	8	164	100/100	93
N11	0.0063	−1.28	GPX1	IPI00319652	14775	22553.4	6.74	7	457	100/100	404
M17	0.027	−1.28	COTL1	IPI00132575	72042	16048	5.28	9	318	100/100	244
M12	0.013	−1.28	PRDX2	IPI00117910	21672	21936.1	5.2	9	593	100/100	515
M14	0.029	−1.26	PARK7	IPI00117264	57320	20236.5	6.32	9	334	100/100	261
M3	0.011	−1.25	PDIA3	IPI00230108	14827	57098.9	5.88	28	692	100/100	426
N17	0.052	−1.24	HMGB1	IPI00420261	15289	25049.2	5.62	11	220	100/100	144
M19	0.016	−1.24	SH3BGRL3	IPI00127358	73723	10527.3	5.02	6	274	100/100	214
M13	0.00047	−1.23	CMPK1	IPI00331146	66588	26040.4	8.13	14	511	100/100	394
N5	0.032	−1.22	RBM3	IPI00130883	19652	16594.7	6.84	6	158	100/100	114
N10	0.021	−1.2	ARPC5L	IPI00111117	74192	17026.8	6.32	7	384	100/100	326

## Supplementary Materials

The following are available online at https://www.mdpi.com/2076-393X/8/1/48/s1, Table S1. Primers used for qRT-PCR; Figure S1. Heatmap of verified genes in microarray analysis; Figure S2. Heatmap of gene sets on “adaptive immune response (GO: 0002250)” using gene set enrichment analysis (GSEA).
