# Supplementary material for: Mechanisms of Mixed Th1/Th2 Responses in Mice Induced by Albizia julibrissin Saponin Active Fraction by in Silico Analysis
Source: Vaccines (Basel). 2020 Jan 27;8(1):48. doi: 10.3390/vaccines8010048 (PMC7158666; doi:10.3390/vaccines8010048)
Supplement: Supplementary file 1 [file vaccines-08-00048-s001.pdf]

vaccines

MDPI

Supplementary Materials

Mechanisms of mixed Th1/Th2 responses in mice induced by *Albizia julibrissin* saponin active fraction by *in silico* analysis

Jing Du, Junjie Jin, Juanjuan Wang and Hongxiang Sun

Table S1. Primers used for qRT-PCR.

Gene	EntrezGeneID	Accession	Primer sequence	Size/bp
GAPDH	14433	NM_008084.3	5′-AGCCTCGTCCCGTAGACAA-3′	104
			5′-AATCTCCACTTTGCCACTGC-3′	
S100A8	20201	NM_013650	5′-GGAGTTCCTTGCGATGGTGA-3′	78
			5′-GGCCAGAAGCTCTGCTACTC-3′	
MCP-1/CCL2	20296	NM_011333	5′-GTCTGTGCTGACCCCAAGAAG-3′	62
			5′-TGGTTCCGATCCAGGTTTTTA-3′	
IFN-γ	15978	NM_008337	5′-TCTTGAAAGACAATCAGGCCATCA-3′	233
			5′-GAATCAGCAGCGACTCCTTTTCC-3′	
T-bet	57765	NM_019507	5′-ATTGGTTGGAGAGGAAGCGG-3′	129
			5′-GCACCAGGTTCGTGACTGTA-3′	
FAM19A3	329731	NM_183224	5′-AGTAGCGAGAACGACTCCCA-3′	132
			5′-TCCGGCTTAGAGCACCTAGA-3′	
IL-5	16191	NM_010558	5′-GCTGGCCTCAAACTGGTAATGTA-3′	100
			5′-GGCAATGGTGCATGTCTGTAACCTC-3′	

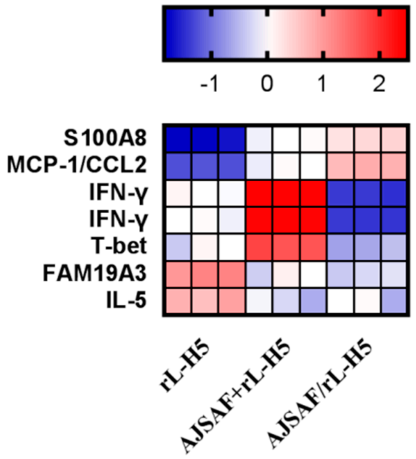

Figure S1. Heatmap of verified genes in microarray analysis.

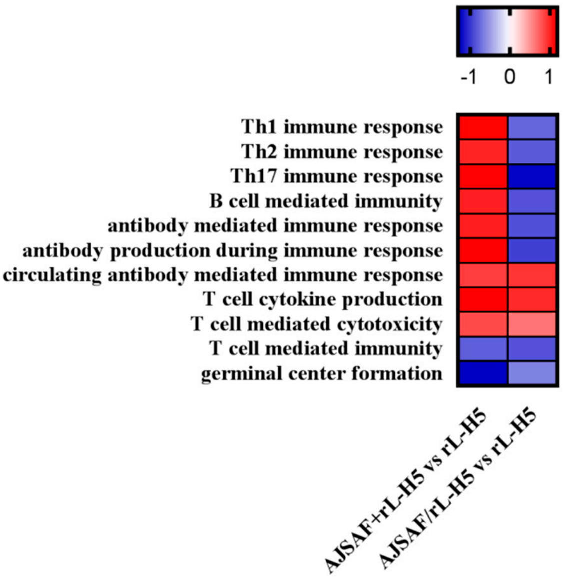

Figure S2. Heatmap of gene sets on "adaptive immune response (GO: 0002250)" using gene set enrichment analysis (GSEA).

© 2020 by the authors. Licensee MDPI, Basel, Switzerland. This article is an open access article distributed under the terms and conditions of the Creative Commons Attribution (CC BY) license (http://creativecommons.org/licenses/by/4.0/).
